# The Mediating Role of Depression in the Effect of Psychological Well-Being on the Self-Rated Health and Quality of Life of Older Adults: Cross-Sectional Study

**DOI:** 10.2196/57731

**Published:** 2025-11-04

**Authors:** Meng Zhang, Ping Zhang, Qiu Xiang, Xiaopan Li, Suqin Xu

**Affiliations:** 1 Nursing Department, Tongji Hospital affiliated to Tongji Medical College of Huazhong University of Science and Technology, No. 1095 Jie Fang Avenue, Hankou, Wuhan, P.R. China, 430030, China, 86 02783665521

**Keywords:** aged, depression, health, quality of life, psychological well-being

## Abstract

**Background:**

A growing body of evidence has identified that people’s physical health could influence self-rated health and quality of life (QoL). However, only focusing on physical health is not adequate for the well-being of older adults. Studies focusing on the impact of psychological well-being on self-rated health and QoL are still rare.

**Objective:**

This study aimed to identify the mediating effect of depression on the association between psychological well-being and self-rated health and QoL to comprehensively understand the relationship between them.

**Methods:**

We used a cross-sectional study design and secondary data analysis from the Chinese Longitudinal Healthy Longevity Survey of 2017 to 2018. Path analysis was applied to examine the research questions.

**Results:**

A large sample of 8839 older adults was included. Among them, more positive affect was found among those who were younger and had more years of schooling, higher household income, greater social security and social insurance, lower depression levels, and higher self-rated health levels. Depression had a partial mediation effect of psychological well-being on self-rated health and QoL, which explained 36% of the total variance (*R*^2^=0.36). In addition, psychological well-being had a statistically significant direct effect on self-rated health and QoL (β*=*0.290; *P*<.001).

**Conclusions:**

Our results indicate that psychological well-being had both direct and indirect effects on self-rated health and QoL. Depression was an important mediator that regulated the effect pathway in older adults.

## Introduction

Populations are aging all over the world. The United Nations General Assembly announced in May 2020 that 2021 to 2030 would be the decade of healthy aging, emphasizing the need for worldwide policymakers to concentrate their efforts on enhancing the lives of older people now and in the future [1]. It is projected that the number of older adults worldwide will rise by approximately 20.6% by 2050, reaching an estimated 2 billion [2]. China has the largest population of older adults [3]. The percentage of Chinese citizens over 60 years of age is expected to reach 28% by 2040 [4]. The second baby boomers, those born between 1962 and 1975, will begin to retire in 2022, adding to China’s aging problems [1].

Depression has become a prevalent mental health symptom among older adults [5,6]. Depression can be divided into clinical and nonclinical subtypes [7]. The clinical form, formally termed major depressive disorder, is a severe psychiatric syndrome marked by enduring dysphoria, anhedonia, and pervasive hopelessness [7]. In contrast, nonclinical depressive states exhibit milder symptoms and are usually precipitated by specific stressors or life events [7]. Community surveys indicate that the global prevalence of nonclinical depression in older adults ranges from 28.4% to 35.1%, whereas prevalence ranges from 27% to 37.3% in mainland China [5,6]. Epidemiological data further show that the frequency of clinical depression among older adults is 1.6% in the United States and 2.7% in China [8].

The course of depression in older adults tends to be more prolonged, and the therapeutic outcomes are generally less favorable compared to those of younger individuals [9]. Depression lowers quality of life (QoL) and raises the mortality risk of older adults [10]. In one study, compared to those without depression, older adults with depression reported lower QoL [11]. A worse overall QoL was linked to a higher degree of depression regardless of the method used to measure QoL. The connections seemed to be steady over time [11].

Focusing on the psychological well-being of older people is essential to constructing a positive aging society and realizing the strategic goal of healthy aging. Healthy aging is defined as the process of developing and maintaining the functional ability that enables well-being in older age [12]. Self-rated questions are frequently used to evaluate health status and QoL, which are essential components of healthy aging [13,14].

Self-rated health (SRH) is the subjective assessment of an individual’s current state of health. It is a valid and reliable measure among those without cognitive impairment [15]. Despite its seeming simplicity and subjectivity, SRH is an effective health indicator that predicts future health outcomes, including mortality, chronic disease incidence, and health care resource use [16]. SRH has also been shown to be stable across cultures, communities, and different age groups, and it has even been shown to be a more accurate predictor of all-cause death than tools created especially for this purpose [17]. A growing body of research reports that even after adjusting for demographic, social, and medical risk variables, SRH has been repeatedly demonstrated to be an independent predictor of mortality, exhibiting high reliability and validity as well as predictive value across a variety of health outcomes [17-19].

Self-rated QoL is one of the main elements that indicate healthy aging. QoL refers to the evaluation of the positive aspects of one’s own life [20]. It is closely associated with social well-being and overall health; however, it is common for older adults to experience a decline in QoL as they age [20]. A recent study discussed a well-being paradox phenomenon that older adults with higher levels of SRH have worse health based on objective markers [21]. Another study pointed out that older adults may mitigate the disparity between potential and actual circumstances through the recalibration of expectations and internal benchmarks [22]. Therefore, they may experience improved QoL even if there are no appreciable health effects.

Prior studies have demonstrated that depression and anxiety mediate the relationship between social and biological factors and QoL [23]. Another longitudinal study indicated that increased depressive symptoms are associated with reduced SRH among Chinese individuals aged ≥45 years [24]. Most existing studies have primarily concentrated on social, biological, and psychopathological factors (depression and anxiety) as key determinants of SRH and QoL [23,24], thereby neglecting the underlying mechanisms that explain the connections between psychological variables (positive and negative affect) and SRH and QoL [25,26]. The mediating role of the main psychopathological factor (depression) in the relationship between psychological well-being and SRH and QoL remains unclear.

According to the hierarchy of needs theory by Maslow [27], humans will pursue higher-level needs only after their lower-level needs have been satisfied. We hypothesize that positive psychological well-being and the absence of emotional disorders are essential to the pursuit of self-actualization to obtain high SRH and QoL. The assumed relationships were that psychological well-being is positively associated with SRH and QoL, while depression is negatively associated with SRH and QoL. Based on this theory, we explored whether depression was a potential mediator of the association between psychological well-being and SRH and QoL in older people, and we applied further path analysis to clarify its potential mechanism. By examining this mediating effect, we can gain a comprehensive understanding of the complex interplay between these variables and potentially identify targets for intervention to improve the health and QoL of older adults. We obtained the data from a representative national survey and analyzed the statistics using path analysis. Our efforts may provide new insights into the mediating role of psychological well-being in promoting SRH and QoL in older people. We also hope to contribute to the body of knowledge on psychological well-being and its implications for the nursing care of older populations, ultimately aiming to promote positive aging.

## Methods

### Data and Study Design

The Chinese Longitudinal Healthy Longevity Survey (CLHLS) was launched in 1998 and continued through waves conducted in 2000, 2002, 2005, 2008-2009, 2011-2012, 2014, and 2017-2018 [28]. This cross-sectional study used data from the 2017-2018 survey wave of the CLHLS to undertake a secondary analysis. The CLHLS national survey was carried out in a randomly selected half of the counties and cities across 22 of the 31 provinces in China, which account for approximately 85% of the country’s total population [28]. Nine provinces were excluded from the CLHLS, all located in western and northwestern China, where minority populations are comparatively large and age reporting tends to be less accurate [28]. The eligibility criteria for the CLHLS study were that the participants be aged 65 years or older [28]. The CLHLS tried to interview all centenarians who voluntarily agreed to participate in the study in the sampled counties and cities [28]. A targeted random sampling method was adopted to ensure the representativeness of the sample [28]. Specifically, it involved interviewing approximately equal numbers of men and women in their 90s, 80s, 70s, and 60s who lived in proximity to the centenarians [29]. We combined the data with complete statistics of demographics, a psychological well-being scale, the Center for Epidemiologic Studies Depression (CES-D) scale, SRH, and self-rated QoL question in the CLHLS survey. After excluding invalid questionnaires, the final sample size was 8839. The sample formula for path analysis was as follows: $n=\frac{Z^{2}P(1-P)}{d^{2}}$, where *n* is the sample size, *Z* is a standard normal variate, *P* is the expected prevalence, and *d* represents precision or absolute error [30]. *P* was estimated based on the available literature, and its value ranged from 27% to 37.3% [5,6]. The admissible error (*d*) was set at 5% because the type I error rate of 0.05 was acceptable for the difference between the sample statistics and the population parameter to be no more than 5% of the population parameter with a standard 95% confidence level (*Z*=1.96) [30]. The values used in this calculation were *Z*=1.96, *P*=.27 to .37, and *d*=0.05. Accounting for a 20% loss to follow-up rate, the estimated sample size ranged from 362 to 429. Finally, the minimum sample size in this study was set at 429.

### Variables

#### Independent Variable

Psychological well-being was measured using 7 items from the 1998 CLHLS, including positive affect and negative affect [31,32]. Positive affect was assessed using 4 positive items (optimistic: “Do you always look on the bright side of things?”; conscientious: “Do you like to keep your belongings neat and clean?”; sense of personal control: “Can you make your own decisions concerning your personal affairs?”; a positive attitude toward one’s aging: “Are you as happy as when you were younger?”), and negative affect was assessed using 3 negative items (agitation: “Do you often feel fearful or anxious?”; loneliness: “Do you often feel lonely and isolated?”; perceived loss of self-worth: “Do you feel the older you get, the more useless you are?”), which were rated on a 5-point frequency scale from 0 (“never”) to 4 (“always”) in both cases [28]. After reverse coding the negative items, the sum of the scores for all 7 items ranged from 0 to 28, with a higher score indicating more positive psychological well-being [28]. The Cronbach α reached 0.704 to 0.752 in previous studies, which indicates an acceptable internal consistency [31-34].

#### Mediation Variable

The 10-item CES-D scale was developed by Andresen et al [35] in 1994 for use with older adults who were relatively well. The CES-D is a screening tool to identify individuals and population groups at elevated risk of nonclinical depression [36]. The internal consistency was satisfactory (Cronbach α=0.78-0.79), and moderate consistency over 3 years was also found to be significant (*r*=0.44; *P*<.01) [37]. The CLHLS used Chinese translations available at the Center for Epidemiologic Studies website [28]. Interviewees had 5 answers to choose from in the 10 CES-D items: “never,” “rarely,” “sometimes,” “often,” and “always.” For questions 1, 2, 3, 4, 6, 8, and 9, we scored 3 for “always” and 0 for “never.” For questions 5, 7, and 10, we scored 0 for “always (good)” and 3 for “never (very bad)” [28]. Therefore, CES-D scores ranged from 0 to 30, with 10 and 20 as cutoff points for the levels of depressive symptoms [38].

#### Dependent Variables

SRH, introduced in the 1980s, is a subjective indicator of health status for older adults [39]. It was measured using the following question: “How do you rate your life at present?” [39]. Self-rated QoL is a subjective assessment of QoL by individuals [40]. It was evaluated using the following question: “How would you rate your overall quality of life?” [40]. SRH and QoL were rated on a 4-point scale ranging from 0 (“very bad”) to 4 (“very good”) [39,40]. Higher scores represented better SRH and QoL status. The widely used single-question method has been found to be reliable and valid [13,41,42].

### Data Analysis

SPSS Statistics (version 25.0; IBM Corp) and SPSS Amos (version 23.0; IBM Corp) were used to analyze the data. Continuous variables were represented using means and SDs or medians and IQRs, whereas categorical or ranked variables were represented using frequencies and percentages. The chi-square test was conducted to assess categorical data, and the Kruskal-Wallis test was conducted to evaluate continuously ranked data. The Spearman correlation was used to examine the relationship among sociodemographic variables, depression, SRH, and QoL. The hypothesized model was tested using the maximum likelihood method in the SPSS Amos statistical software. Several model fit indices were estimated to test the good fit of the model, including the chi-square test, root mean square error of approximation (RMSEA), standardized root mean square residual (SRMR), cumulative fit index (CFI), incremental fit index, goodness-of-fit index (GFI), normed fit index (NFI), and Tucker-Lewis index (TLI). A good fit model was considered when all path coefficients were significant at the level of .05, *χ*^2^<5, SRMR<0.08, RMSEA<0.08, GFI>0.9, NFI>0.9, TLI>0.9, and CFI>0.9 [43]. All analyses were 2-tailed.

### Ethical Considerations

The CLHLS study was approved by the Duke Health Institutional Review Board (Pro00062871) and the Research Ethics Committee of Peking University (IRB00001052-13074), and all participants provided written informed consent. The data were made publicly available for secondary analysis after being fully anonymized. No compensation was provided to participants.

## Results

### Participant Demographics

**Figure 1.**
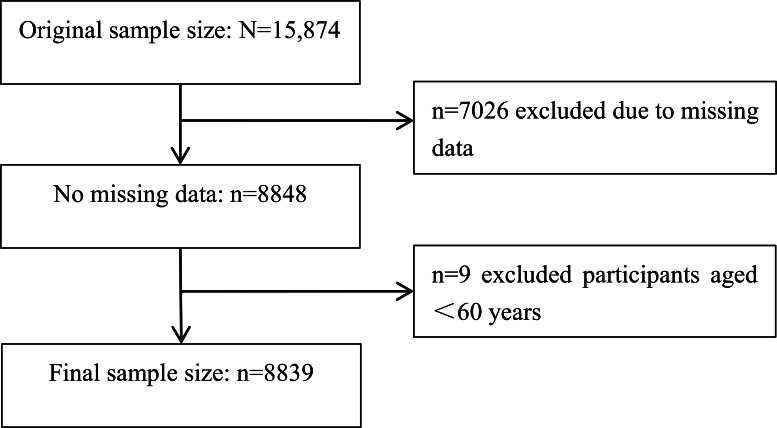
Flowchart of the sample selection.

A total of 8839 older adults were included in this study (Figure 1), with 48.7% (4305/8839) being male and 51.3% (4534/8839) being female. The average age was 81.27 (SD 10.29) years, and they had a median of 3 (IQR 0-6) years of schooling. The median total income in the previous year was ¥30,000 (US $4212.57; IQR ¥8000 to >¥99998 [US $1123.35 to >US $14,041.62]). Approximately half (4384/8839, 49.6%) of the participants were currently married or living with a partner. More than 90% of the participants (8140/8839, 92.1%) had social security or social insurance. The average scores for depression, SRH, and QoL were 9.62, 2.51, and 2.91, respectively. A total of 52.1% (4607/8839) of the participants scored lower than 10 in the depression scale, 45.5% (4028/8839) scored between 10 and 20, and 2.3% (204/8839) scored higher than 20.

### Variables According to Different Psychological Well-Being Levels

A substantial difference was observed in age, sex, years of schooling, total household income in the previous year, social security and social insurance status, depression, QoL, and SRH (Table 1). Younger participants and those who had more years of schooling, higher household income, social security and social insurance, lower depression levels, and higher SRH and QoL levels indicated more positive affect. Female participants had higher positive affect levels than male participants.

**Table 1.** Variables according to different psychological well-being levels. Psychological well-being was divided into 3 levels based on tertile range.

Variable	Psychological well-being level			Statistic	
	More negative (score of <19; n=3832)	Moderate (score of 19-21; n=2124)	More positive (score of >21; n=2883)	Kruskal-Wallis *H* statistic	Chi-square (*df*)
Age (y), mean (SD)	81.72 (10.32)	80.77 (10.29)	81.02 (10.23)	13.72^a^	—^b^
Sex (female), n (%)	2063 (53.84)	1023 (48.16)	1448 (50.23)	—	19.6^c^ (2)
Years of schooling, median (IQR)	2 (0 to 6)	3 (0 to 6)	4 (0 to 8)	76.55^c^	—
Total income in the previous year, median (IQR)	30,000 (US $4212.57; IQR ¥6000 to 80,000 [US $842.51 to US $11,233.52])	35,000 (US $4914.67; IQR ¥9000 to 99998 [US $1263.77 to US $14,041.62])	40,000 (US $5616.76; IQR ¥10,000 to 99998 [US $1404.19 to US $14,041.62])	54.37^c^	—
Social security and insurance (yes), n (%)	3490 (91.1)	1959 (92.23)	2691 (93.34)	—	9.5^a^ (2)
Depression, mean (SD)	12.36 (1 to 29)	9.16 (1 to 28)	6.32 (1 to 26)	2578.38^c^	—
QoL^d^, mean (SD)	2.68 (1 to 4)	2.92 (1 to 4)	3.21 (1 to 4)	773.07^c^	—
Self-rated health, mean (SD)	2.23 (1 to 4)	2.53 (1 to 4)	2.86 (1 to 4)	955.07^c^	—

a *P<*.01.

b Not applicable.

c *P<*.001.

d QoL: quality of life.

### Correlation of Variables

Table 2 shows the correlation among psychological well-being, depression, SRH, and QoL. The Spearman correlation suggests that psychological well-being had a negative correlation with depression (β*=*–0.590) but psychological well-being had a positive correlation with QoL (β=0.326) and SRH (β=0.361). Moreover, depression was significantly negatively correlated with QoL (β=–0.354) and SRH (β=–0.382). In addition, QoL was significantly positively correlated with SRH (β=0.463).

**Table 2.** Spearman correlation of variables.

Variable	Psychological well-being (*r)*	Depression (*r)*	QoL^a^ (*r)*	Self-rated health (*r)*
Psychological well-being (*r)*	1	—^b^	—	—
Depression (*r)*	−0.590^c^	1	—	—
QoL (*r)*	0.326^c^	−0.354^c^	1	—
Self-rated health (*r)*	0.361^c^	−0.382^c^	0.463^c^	1

a QoL: quality of life.

b Not applicable.

c *P<*.001.

### Mediator Model

**Figure 2.**
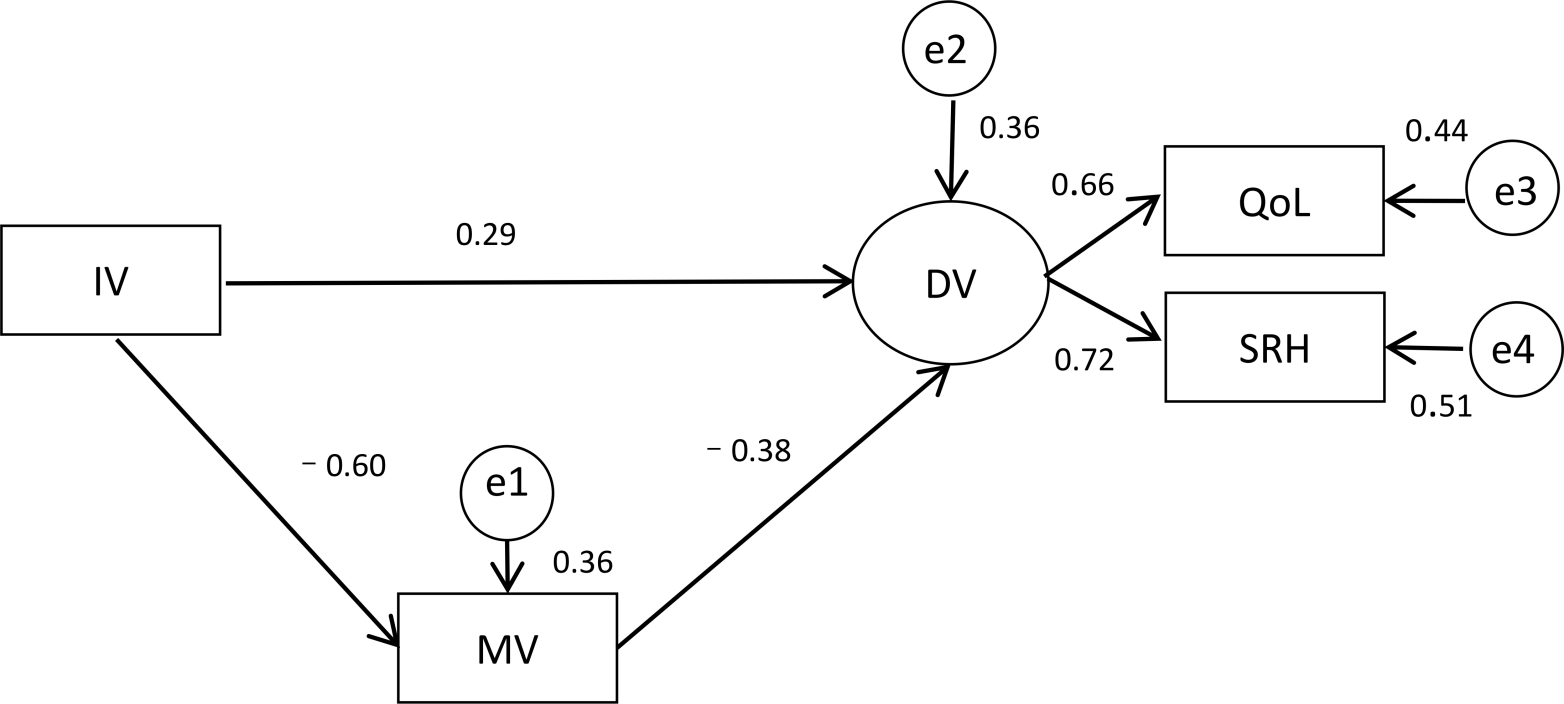
Mediator model. DV: dependent variable (quality of life [QoL] and self-rated health [SRH]); e: the residual of the variable; IV: independent variable (psychological well-being); MV: mediation variable (depression).

Figure 2 illustrates the standardized effects of the coefficients in the mediator model. All path estimates were statistically significant (*P*<.001) and correlated with the hypothesized model. Depression had a partial mediation effect of psychological well-being on SRH and QoL, which explained 36% of the total variance (*R*^2^=0.36). Psychological well-being had a statistically significant negative direct effect on depression (β=−0.603; *P*<.001), whereas depression had a further statistically significant negative direct effect on SRH and QoL (β=–0.378; *P*<.001). In addition, psychological well-being had a statistically significant positive direct effect on SRH and QoL (β=0.290; *P*<.001). Furthermore, the indirect effect of psychological well-being on general health was β = (−0.603) × (−0.378) = 0.228. In addition, SRH (β=0.716) contributed more to general health than QoL (β=0.663).

Fit indexes of the mediator model revealed a good fit of the data (*χ*^2_1_^=1.7; *P*=.19). The SRMR was 0.006, and the RMSEA was 0.009, which indicated good validity of the measurement construct. The scores of the GFI, NFI, TLI, CFI, and incremental fit index were over 0.9, further illustrating the good model fit.

## Discussion

### Principal Findings

Our study found that psychological well-being had a negative direct effect on depression, whereas depression had a further negative direct effect on SRH and QoL. In addition, psychological well-being had a positive direct effect on SRH and QoL. The results were consistent with those of previous research that highlighted the effect of psychological well-being and depression on SRH and QoL [33]. Moreover, the results also illustrated that younger older adults who had more years of schooling, higher household income, and greater social security and social insurance indicated more positive affect. These findings expand those of previous studies by exploring the mediating role of depression among older adults in China, thus providing a reference for improving the psychological well-being of Chinese older adults.

Psychological well-being was regulated by older people’s demographics, such as age, sex, educational background, and income. A very interesting finding was that people aged >81 years tended to have more positive affect or more negative affect. This could be explained by the “response shift” phenomenon, in which some older adults reconcile with the deterioration of health and functionality [22]. This reconciliation often involves the recalibration of expectations and internal benchmarks to mitigate the disparity between potential and actual circumstances through both biological and psychosocial transformations [22]. Our study found that women had more negative affect than men. The reason may be that women have more negative reactions when encountering negative stimuli in comparison to men [44]. The results also demonstrated that total income, years of schooling, and social security and insurance status were positively associated with psychological well-being. Previous studies have proved the modest correlations between socioeconomic status, such as income, educational level, and occupation, and personal psychological well-being, which is consistent with the results of our study [45].

This study indicated that positive affect decreased the incidence of depression and enhanced the SRH and QoL of older adults. Our results were aligned with those of a previous study [33] and provided a specific pathway and coefficient statistics among variables of psychological well-being, depression, SRH, and QoL. Psychological well-being is very important to an individual and may predict the occurrence of late-life depression in older adults. The older adults who had more positive affect were relatively more likely to look at the bright side of the world and focus on the goodness of things [46]. In positive psychology theory, the novel ideas and actions (eg, the urge to play and explore) brought by broadened mindsets have been proven to alleviate depression and improve general health [47].

The path analysis revealed that depression was a mediator of the effect of psychological well-being on SRH and QoL in older adults, which accounted for 36% of the variance. The size of its indirect effect of psychological well-being on SRH and QoL (β=0.228) was very close to the direct effect size (β=0.290), which implied that depression was an important mediator between psychological well-being and SRH and QoL. Depression is a mental health symptom and has adverse consequences for SRH and QoL in older adults. Approximately half (4232/8839, 47.9%) of the older adults in this study had CES-D scores of 10 and over, higher than the incidence in Indonesia and previous samples in China, such as the 2011 to 2012 wave [48]. Our findings are consistent with the hypothesis that depressive symptoms among Chinese older adults have risen in recent years [49]. Nevertheless, focusing on the psychological well-being of older adults is urgent to promote healthy aging.

Paying attention to psychological well-being and reducing depression may be an effective strategy for promoting the health and QoL of older people. Previous studies have indicated that people with depression show specific deficits in being able to anticipate future positive events [50]. The ability to think mindfully, develop a tolerant outlook, please oneself, or cope when confronting situations that lead to negative affect may be powerful measures to construct healthy psychological well-being. We should also focus on the support systems of older adults, which serve as a protective factor for psychological well-being to promote the health of older adults [51].

China has the fifth highest older adult population worldwide, which is frequently cited as an example for other middle-income nations [52]. Our results could also have implications for limited-income countries. Future research could involve additional countries to compare the influence of various cultures and health care systems on older adults’ psychological well-being, health, and QoL.

### Limitations

Although our data were derived from a large Chinese population-based older adult cohort, there were still some limitations to our study. First, the cross-sectional design could not explain the causal effects of the variables when compared with longitudinal studies. These results may not be temporally stable over time. Second, the participants may have provided biased responses due to memory recall issues when the data were collected. Third, our study only examined the relationships between 4 indicators: psychological well-being, depression, SRH, and QoL. If more indicators had been included, the conclusions may have been richer. Fourth, the analysis was based on self-reported questions on depression, well-being, and QoL. Moreover, the CES-D is a screening tool, not a clinical diagnostic tool.

### Conclusions

Our study provides a new insight into the influence of the psychological aspect on older adults’ health and QoL. We also discussed the variable of depression, a very common type of emotional distress, in the mediation pathway between psychological well-being and SRH and QoL. Depression was an important mediator of the effect of psychological well-being on SRH and QoL in older people. More attention should be paid to depression and the psychological well-being of older adults. Future tailored interventions should focus on relieving depression in older adults and promoting their psychological well-being.

## Abbreviations

CES-D: Center for Epidemiologic Studies Depression
CFI: cumulative fit index
CLHLS: Chinese Longitudinal Healthy Longevity Survey
GFI: goodness-of-fit index
NFI: normed fit index
QoL: quality of life
RMSEA: root mean square error of approximation
SRH: self-rated health
SRMR: standardized root mean square residual
TLI: Tucker-Lewis index
